# Supplementary material for: Structural and Functional Analysis of SHP Promoter and Its Transcriptional Response to FXR in Zn-Induced Changes to Lipid Metabolism
Source: Int J Mol Sci. 2022 Jun 10;23(12):6523. doi: 10.3390/ijms23126523 (PMC9224202; doi:10.3390/ijms23126523)
Supplement: Supplementary file 1 [file ijms-23-06523-s001.zip › ijms-1758792-supplementary.pdf]

Table S1. Primers used for SHP promoter cloning.

Gene	Forward primer (5'-3')	Reverse primer (5'-3')	Step
SHP	ACGATGGACTCCAGAGCGG CCGCVVNVNNNCCAA	GATGTTGAACAGGATGG CGTGTGC	hiTAIL -PCR1
	ACGATGGACTCCAGAG	CATGCGTAAGAGACCGG ATGTGCGT	hiTAIL -PCR2
	ACGATGGACTCCAGAG	ACGCACATCCGGTCTCTT ACGCAT	hiTAIL -PCR3

Table S2. Primers used for 5’-deletion plasmids construction.

Gene	Primers	Forward primer (5'-3')	Reverse primer (5'-3')
SHP	pGl3- -346/+48	gaccggtaccgagctcTTAAAGAA CTTTTTAACATATCAAC	agtaccggaatgccaagcttTCTCTTA CGCATGAGACTGCAGGCT
	pGl3- -808/+48	gaccggtaccgagctcACCCTGTC ACCCTAACCCTGTAACC	agtaccggaatgccaagcttTCTCTTA CGCATGAGACTGCAGGCT
	pGl3- -1252/+48	gaccggtaccgagctcCTAACCCT GTAACCCTGTAACCCTG	agtaccggaatgccaagcttTCTCTTA CGCATGAGACTGCAGGCT
	pGl3- -1783/+48	gaccggtaccgagctcAACCTCTC TAGGGGGAACCCCCTAA	agtaccggaatgccaagcttTCTCTTA CGCATGAGACTGCAGGCT

Table S3. The reference binding site sequences.

Name	Binding site sequences
ERRγ	AAGCTCA
FXR	AGGTCA
KLF4	AAAGGAAGG
PPARα/RXR	GGNAAAGGT
Sp1	CCCCNCCCC
SREBP1	TCACCCA
STAT3	TTCTNGGAA
TATA-box	TATAAA
NF-Y	CCAAT

Table S4. Primers used for site-mutation analysis.

Gene	Primers	Forward primer (5'-3')	Reverse primer (5'-3')
SHP	Mut-FXR-1	TGTTatctactggtGTGTTACAT TAAATCACCTTAAATTCTG TT	ACACaccagtagatAACATTT ACACACTGAATGTTAAT GTTCA
	Mut-FXR-2	ATCTtgggtaagcaGCAGGGTC ATTGACCTTTCTGT	CTGCtgcttacccaAGATATA ACACACACACACACACA CACA

Table S5. Primers used for electrophoretic mobility-shift assay.

Primers		Forward primer (5'-3')	Reverse primer (5'-3')
SHP -FXR-1	Biotin-probe	Biotin-ATGTTAGAGGTCAGTGTGTT	Biotin-AACACACTGACCTCTAACAT
	Mutative-competitor	ATGTTATCTACTGGTGTGTT	AACACACCAGTAGATAACAT

Table S6. Primers used for Q-PCR analysis.

Gene	Forward primer (5'-3')	Reverse primer (5'-3')
6PGD	CTGCTGCTGGACTCCTTCTT	GTTGTGTCTGTAACCGTCGTAA
β-actin	GTGCGTGACATCAAGGAGAAG	CGAGGAAGGATGGCTGGAA
ACCα	ACTTCTGCTGTGGTTGTCCTAT	GCATCCATCGTGGGTCATA
ATGL	ATCTGGTGAAGGTGCTGAAGT	CTCTGACTGTGGCAGGTTGT
CPT Iα	CGCTCCTGCTCCAATGAGA	GAGACCACATAGAGGCAGAAGA
FAS	CATCATCACTGGAGGTCTTGGA	TACGAATGCCTGATCTGGAAGT
FXR	ATGCTTGCGGAATGTTTACT	TTGTGGATGTGACCTGCTTAG
G6PD	GAGAAGCCTGCCTCAACCA	GGATCGTCCAAGTAGCCAAGT
HSL	ACCATTGCTCCACCGTCTG	CGTCTCACTATCCTGTCCTTCA
SHP	CGCACAAGAGCACGTAACAT	CAAGCCTCCAGAGTTTGTCC
